# Topological guiding of elastic waves in phononic metamaterials based on 2D pentamode structures

**DOI:** 10.1038/s41598-017-18394-8

**Published:** 2017-12-22

**Authors:** Yuning Guo, Thomas Dekorsy, Mike Hettich

**Affiliations:** 1 Department of Physics, University of Konstanz, 78457 Konstanz, Germany; 2 Institute of Technical Physics, German Aerospace Center, Pfaffenwaldring 38–40, 70568 Stuttgart, Germany

## Abstract

A topological state with protected propagation of elastic waves is achieved by appropriately engineering a phononic metamaterial based on 2D pentamode structures in silicon. Gapless edge states in the designed structure, which are characterized by pseudospin-dependent transport, provide backscattering-immune propagation of the elastic wave along bend paths. The role of the states responsible for forward and backward transfer can be interchanged by design.

## Introduction

Topological insulators are observed in electronic systems and are based on the coexistence of counter-propagating spin-polarized edge states^1^. This observation has inspired a search for analogous protected edge states in non-electron based systems. The topological properties of classical waves with non-reciprocity in bosonic systems based on photonics^2–4^, acoustics^5–9^, and mechanics^10–13^ have made a flourishing development in recent years. In addition, magnetization twisting skyrmions, which also have non-trivial topological properties, have received considerable attention^14,15^. In general, the development of topological materials is based on the concept of the Dirac point and the analogy of the quantum spin Hall effect associated with the theoretical analysis of the Berry phase.

As edge states of topological insulators possess the ability to propagate along a specific direction without backscattering, energy can be confined or guided on the surface or along the interface/boundary of these materials. The key feature of the topological electronic model was transferred to the field of photonics in photonic crystals first in 2005^4^, which spurred numerous subsequent theoretical and experimental investigations in bosonic systems. A topological photonic insulator was realized in a composite lattice structure by a zone folding mechanism^2,16^. Recently topological properties are not only studied in photonics, but also in phononics. The edge modes in phononic systems are important in many aspects including vibration control and acoustic imaging^17–19^. By using a perturbation method, the double Dirac cone with the occurrence of accidental degeneracy was obtained in core-shell-structure phononic crystals^20^. A convenient way to obtain acoustic topological states was adopted by dynamically modulating the parameters of inclusions^5,21^. The topological nontrivial phase was realized by breaking time-reversal through designing an on-site rotating modulation scheme in a Floquet topological insulator^6^, setting rotating fluids into the “meta-atom”^7,22^ or breaking inversion symmetry through utilizing chiral interlayer coupling^23^. A study confirmed that symmetry-protected 3D topological bandgaps supporting disorder-immune surface states can be obtained in bosonic systems^24,25^. Elastic waves including Lamb waves also exhibit properties related to topological states. With the Coriolis forces present in gyroscopes, chiral edge modes of elastic waves were obtained in a hexagonal lattice^12^. Topologically protected elastic waves were realized in both static and time-dependent regimes by designing a spin-degenerate metacrystal in a dual-scale phononic crystal slab^13^. In addition, the topological phase of sound and light based on cavity optomechanics was demonstrated, which gives rise to the possibility to produce a flexible optomechanical concept rather than purely geometry-based approaches^8^. Given a large number of recent works on topological phononics, we expect to see the appearance of many works leading the way to new applications regarding phonon guiding from the sonic to the thermal range. To obtain topologically protected edge states, the symmetries of spatial inversion and time-reversal have to be broken, then nonzero Berry curvatures and nonzero Chern numbers can be achieved, respectively. By designing structures with a domain wall supporting chiral or helical edge states, the topologically protected transport will emerge at the boundary according to the bulk-boundary correspondence principle^26^.

Metamaterials are engineered structures that realize devices with extraordinary responses to light, sound, and heat flow^27–30^. Phononic metamaterials in solid state systems, which can be termed as elastic metamaterials are used to break or buckle in order to dissipate mechanical shock energy. This work can lead to active mechanical metamaterials, integrating energy sources, feedback loops *etc* ^31,32^. Pentamode materials, a specific type of mechanical metamaterials, only supports longitudinal polarized elastic waves, since the effective shear modulus is very small relative to the bulk modulus. The materials were proposed years ago and have been realized in the mechanical and acoustic field^33–35^. Mechanical cloaking based on pentamode metamaterials can protect sensitive objects mechanically or even hide them^36^. In this paper, we propose a 2D phononic metamaterial, a kind of phononic graphene, based on a 2D pentamode structure in order to realize topologically protected edge states. In particular, the presented approach requires, contrary to most related recent works, no external fields, i.e., it is completely based on passive components. Similar concepts utilize either Lamb modes^13^ or complex bilayer geometries^37^ while we demonstrate that protected edge transport is possible for in–plane propagation in a single layer structure of silicon. This is a considerable advantage in the attempt to fabricate structures aiming for nanotechnological applications in the high-frequency range and thus is an important step towards the realization of actual devices.

## Results

**Figure 1:**
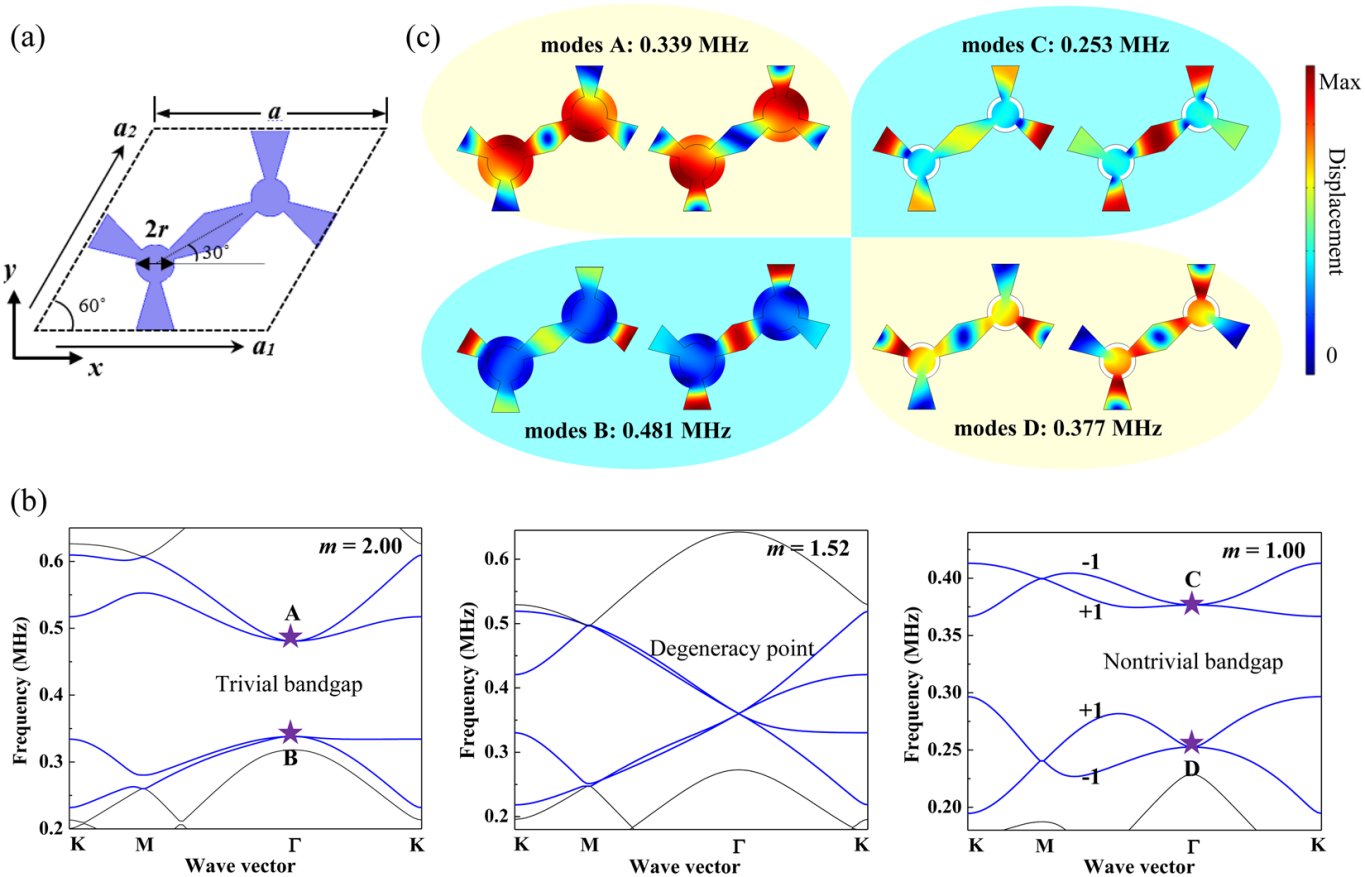
(**a**) The geometry of a unit cell of the 2D pentamode structure with the honeycomb array; (**b**) band structures of the 2D pentamode structure with different radii of joint circle with *m* = 2.00, 1.52, and 1.00, respectively (**c**) the vibrational modes of separated degeneracy points marked in (**b**).

Firstly we introduce the structural parameters of the proposed phononic metamaterial. Figure 1(a) shows the schematic of a unit cell of the 2D pentamode structure with the arrangement of a honeycomb lattice. The radius of a joint circle $r=m\ast {r}_{0}$, where *m* is a scaling factor, and *r*_0_ = 1 mm is defined as the basic value of radius. The lattice constant is given by *a* = 12 mm. We choose silicon as material system with a Young’s modulus E = 167 GPa, a Poisson’s ratio *σ* = 0.27 and a density *ρ* = 2330 kg/m^3^. The modelling software Comsol, based on the finite element method, is employed to study the phononic properties including band structures and transmissions of the designed phononic nanostructures in the paper, while we adopted a finite difference scheme using a skewed 9-point stencil implemented in Matlab to calculate the Berry curvatures. Periodic boundary conditions are set to the four outer boundaries of the unit cell for the band structure calculation. The band structure is calculated by solving the eigenfrequency equation and sweeping the wavevector in the irreducible Brillouin zone of the honeycomb lattice in the reciprocal space. This pentamode material is a kind of elastic metamaterial, however, we like to focus on the novel propagation characteristics of the elastic wave in the pentamode structure, hence we adopt the name of phononic metamaterial here.

We use the geometrical parameter of the joint circle in order to explore different band structures. The band structures of 2D pentamode structures and the accidental degeneracy point are illustrated in Fig. 1(b). A four-fold accidental degeneracy point with linear dispersions appears at the Γ point of the structure with *m* = 1.52. The degeneracy point at 0.360 MHz is a Dirac point with double Dirac cones. The physical origin of a Dirac point at the Brillouin boundary is the consequences of the lattice symmetry, while that of a Dirac point at zone center is the appearance of accidental degeneracy^38,39^. The breaking of time-reversal symmetry can be achieved by separating the Dirac point into two opposite states in the separated bands. With decreasing the parameter of the joint circle *m* from 2.00 to 1.00, the bandgap closes and reopens experiencing a phase transition, i.e., an inversion of band modes take place, which means the trivial bandgap has been transformed into a nontrivial bandgap due to the breaking of time-reversal symmetry. The corresponding calculation of the Berry curvature is shown in supplementary, which demonstrates that the structure with *m* = 1.0 is a topological structure. The Chern number for the four bands of interest is shown in the topological structure. Therefore, the bandgaps that belong to different topological phases can be transferred by appropriately tuning the system’s parameters.

It is demonstrated by the band structures that by modulation of the parameter the designed structures undergo a phase transition in reciprocal space. The analysis of vibrational modes confirms these results in an intuitive way. Figure 1(c) shows the displacement fields of separated degenerated points, which are used to identify the vibrational modes of different structures. The four-fold accidental degeneracy point is separated into two double-fold degeneracy points in both topological and ordinary structures, thus, each double-fold degeneracy point at the Γ point exhibits two vibrational modes at the same frequencies. We can observe from Fig. 1(c) that the vibrational modes of the degeneracy modes A with the frequency of 0.339 MHz in the upper bands of the ordinary structure are the same as the degeneracy modes D with the frequency of 0.377 MHz in the lower bands of the topological structure, i.e., the modes of separated degeneracy points in the upper bands of the ordinary structure with the trivial bandgap are coincident with that in the lower bands of the topological structure with the nontrivial bandgap. The degeneracy modes B at 0.481 MHz in the lower bands of the ordinary structure and the degeneracy modes C in the upper bands of the topological structure demonstrate the same conclusion and confirm the appearance of a phase transition from zero Chern Number to non-zero Chern Number in the two structures.

Since topologically protected gapless edge states can be formed at the domain wall formed from two phononic structures with dissimilar phases, i.e., the topological structure and the ordinary structure, topologically protected waveguides can be constructed for diverse applications^6,8^.

**Figure 2:**
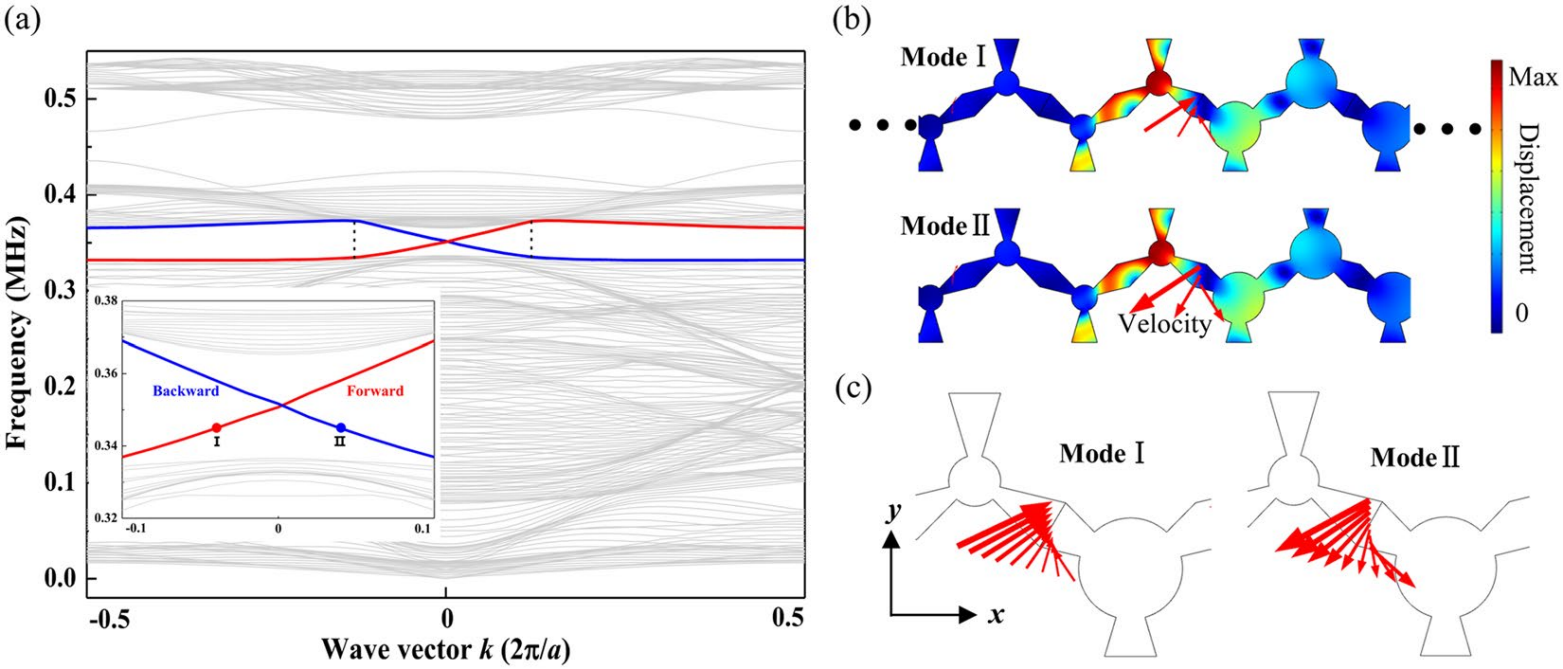
(**a**) The band structure of a supercell consisting of the topological structures stacked with the ordinary structures with helical edge modes, the forward transfer and backward transfer of edge states are marked as red and blue respectively; (**b**) the vibrational modes of the centre region of the stacked structure at *k* =  ± 0.04 (the marked dots in the inset) with a frequency of 0.345 MHz, and (**c**) the corresponding velocity fields at the domain walls. The red arrows represent the amplitudes and the directions of the position dependent velocities.

To confirm the existence of topologically protected edge states, band structure and vibrational mode analysis of a supercell comprising 24 × 1 unit cells is performed numerically. The supercell is a ribbon of 24 unit cells along the *x* direction, which consists of 12 unit cells of the topological structure and 12 unit cells of the ordinary structure in a row. Periodic boundary conditions are applied to the top and bottom edges along *y* directions and free boundary conditions are set on all other edges. Figure 2 shows the band structure of a supercell of the stacked structure and vibrational modes of the edge states with specific wavevectors. The two gapless edge modes, which connect the bulk wave modes, can be observed from the band structure in Fig. 2(a). The local band structure of interest is shown as an inset. Figure 2(b) demonstrates two vibrational modes of the centre region of the stacked structure at 0.345 MHz with the wavevector *k* =  ± 0.04, which shows opposite directions of velocity in the domain wall, i.e., when one is defined as the forward transfer with a positive group velocity, the other one will be the backward transfer with a negative velocity. The corresponding velocity fields of the domain wall, which possess helical characteristics, are unambiguously demonstrated in Fig. 2(c). The two edge branches support modes bound on the interface with opposite velocity directions, i.e., angular momentum, for the counterpropagating waves, which can be treated as a pseudo spin-up state and a pseudo spin-down state analogous to the quantum spin Hall effect in electronic systems. Since the edge bands are located in the gap of bulk waves, the edge modes cannot be scattered into the bulk of the structure theoretically. The helical pseudospin states ensure the absence of backscattering, which will be demonstrated in the analysis of the displacement fields. The vibrational modes of the helical edge states show that the elastic waves are mostly localized in the domain wall and exhibit the respective forward or backward velocity, which is in accordance with the band structure. Besides, some other vibrational modes are checked based on Fig. 2(a). The edge modes inside the area limited by the dashed lines at *k* =  ± 0.12 demonstrate that the vibrational mode and energy concentrate in the domain wall. While the modes located exactly at the wavevector *k* =  ± 0.12 show the hybridization of vibrations of the domain wall and parts of the topological structure or the ordinary structure. When the wavevector is outside of the marked region, the edge modes transform into bulk modes which excite bulk vibrations only. In the following we will investigate different edge geometries and the respective wave propagation behavior.

**Figure 3:**
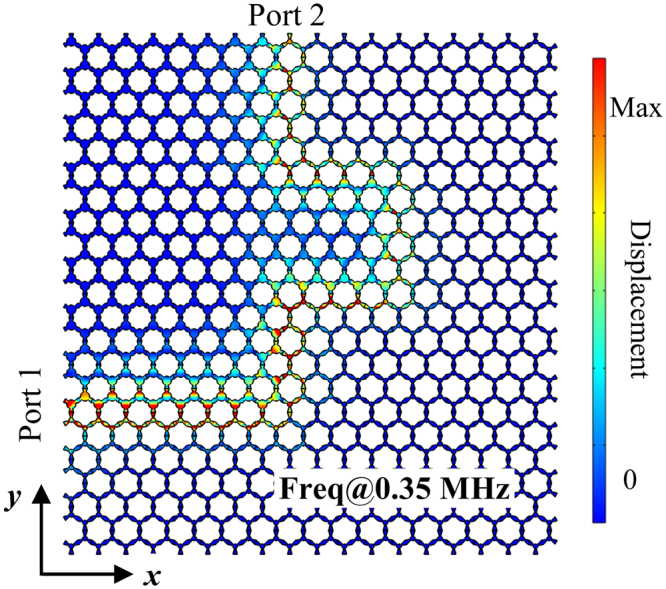
The displacement field of robust propagation of the elastic wave with a frequency of 0.35 MHz along the domain walls in the designed phononic metamaterial consisting of topological structures stacked with ordinary structures. Port 1 and Port 2 indicate different locations of the input.

Figure 3 shows a zigzag domain wall which functions as a curved waveguide in a phononic structure consisting of topological structures stacked with ordinary structures with a frequency of 0.35 MHz. Port 1 and Port 2 indicate different locations of the input. The bend can severely inhibit the wave propagation in ordinary waveguides. However, we can observe that the elastic wave can propagate along the bends, which demonstrates a high-quality transmission in this phononic metamaterial. The robust propagation of elastic waves along the domain wall is ensured by the helical edge modes. The backscattering is largely suppressed in the domain walls due to the topological protection and thus the elastic wave can propagate even around sharp bends in the designed structure. Besides, the simulation results show that no matter if port 1 or port 2 is taken as the incident port, the robust propagation along the domain wall can be obtained. This is a point which will be analyzed in more detail below. Therefore, topologically protected states which are backscatter-immune along zigzag paths can be realized for elastic waves in this type of phononic metamaterial. A prerequisite for actual applications is a deeper understanding of the propagation characteristics and their geometry dependent behaviour. We will discuss this in more detail in the following.

## Discussion

Based on the analysis of the results displayed in Fig. 2, we know that the edge states exhibit forward transfer and backward transfer, which can be treated as pseudo spin-up and pseudo spin-down states, respectively. In order to select a particular pseudospin state from the gapless edge modes, different phononic structures are constructed to study pseudospin-dependent transport.

**Figure 4:**
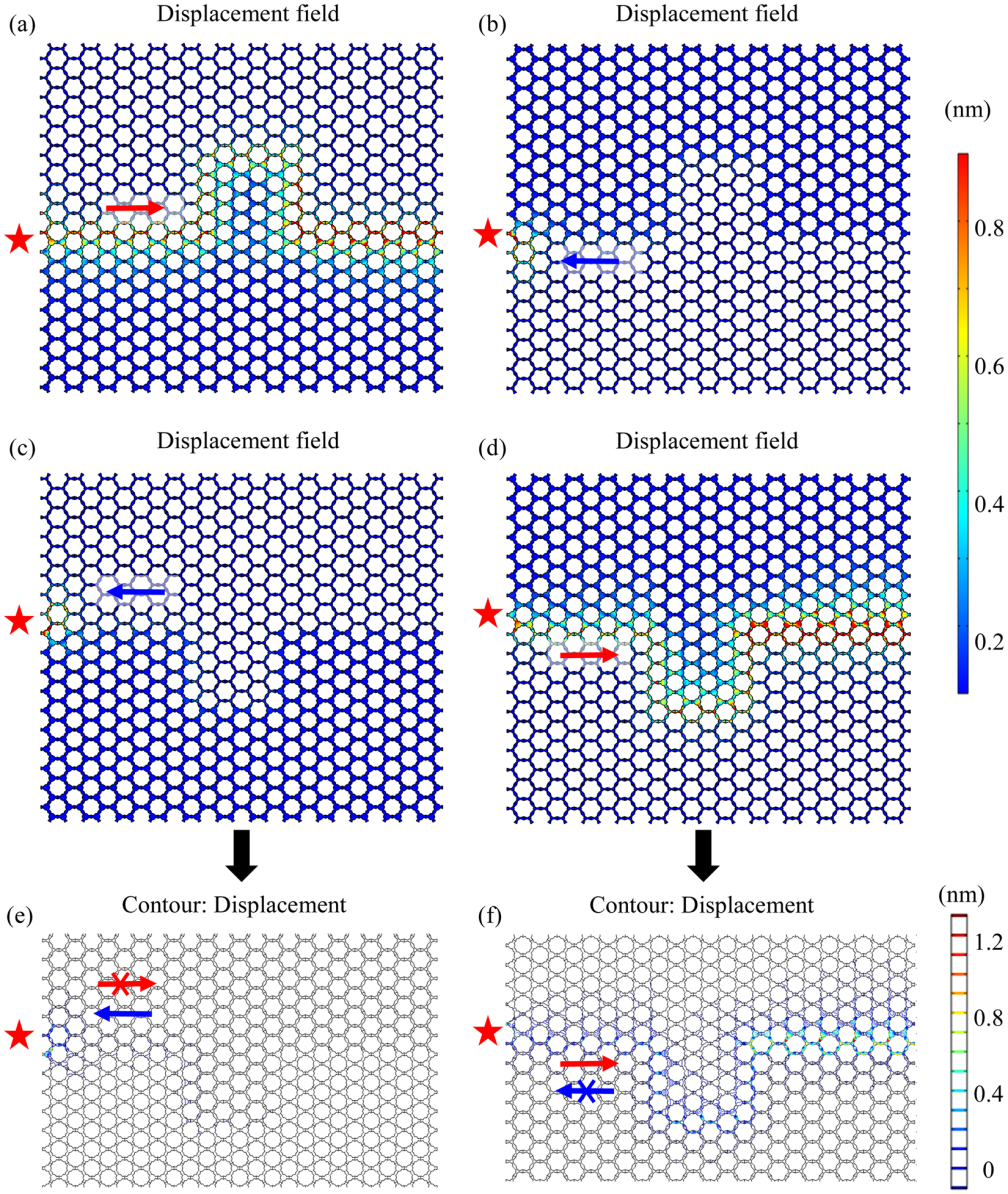
The displacement fields of different phononic structures with a frequency of 0.35 MHz: (**a**) the topological structure coating the ordinary structure with an upward bend; (**b**) the ordinary structure coating the topological structure with an upward bend; (**c**) the topological structure coating the ordinary structure with a downward bend; (**d**) the ordinary structure coating the topological structure with a downward bend; (**e**) and (**f**) are the corresponding displacement contours of (**c**) and (**d**), respectively. The arrows in red and blue represent the forward and backward transport, respectively. The red stars indicate the location of the input.

Figure 4 shows the displacement fields with a frequency of 0.35 MHz of four different phononic structures which consist of the topological structure and the ordinary structure with an upward bend or a downward bend. We find an interesting behaviour comparing Fig. 4(a) with (c) which exhibit an upward and a downward bend respectively. While (a) with the upward bend demonstrates robust transmission, the transmission of the latter with the downward bend is forbidden. Based on Fig. 4(a,b), we observe that even with the same paths for the domain wall and the same input ports, when the location of the topological structure and ordinary structure exchange, the transmission characteristics will change from propagation allowed to forbidden or to the opposite as the direction of velocity changes. The displacement contours of Fig. 4(c,d) demonstrate the effect of the exchange of propagation directions more distinctly. These results prove that the transmission in a specific system, determined by the pseudospin states, is influenced by the configuration of the stacked structures. By comparing Fig. 4(a–c), where the input ports are exchanged, respectively, we found that the characteristic keeps unchanged, i.e., the elastic waves are propagating for (a) & (c) or are forbidden for (b) & (c), which demonstrates that the transmission of elastic waves in our structures is symmetric. Intrinsic absorption can be a limiting factor for the propagation of elastic waves. However, based on the former experimental studies in silicon^40–43^, we can confirm that the absorption length here is much larger than the size of the structure. Thus, the absorption is not expected to be strong enough to prevent the observation of topological modes in the proposed structures.

**Figure 5:**
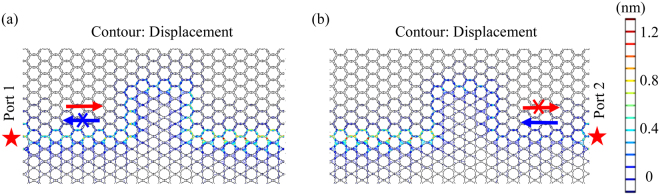
The displacement contours at 0.35 MHz with the input of (**a**) port 1 and (**b**) port 2 in the model described in Fig. 4(a). The red stars mark the incident ports.

Figure 5 shows the displacement contours at 0.35 MHz with different input ports in the model described in Fig. 4(a). The robust propagation along the domain wall can be obtained with both incident ports and the two displacement fields demonstrate the mirror symmetry. The topological edge states obey the symmetry selection rule^13,44,45^ with helical characteristics, which shows the right-handed and left-handed effects on the two sides of the mirror plane for the same energy. Generally, at a specific frequency in the range of helical edge states, the elastic wave can propagate either forward or backward in a structure. Thus, the pseudospin state is known in a specific structure when the location of the excitation source is chosen.

**Figure 6:**
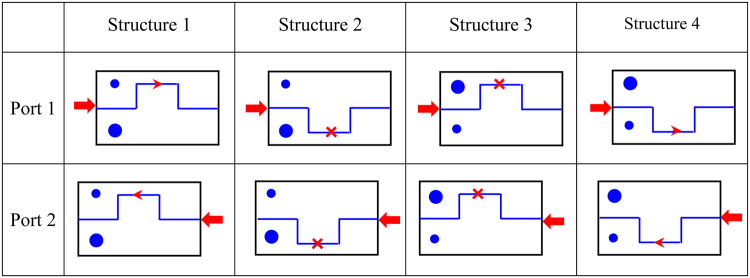
The schematic diagrams of transmissions at 0.35 GHz in different phononic structures. The small circle and larger circle represent a topological structure and an ordinary structure, respectively; the polyline demonstrates the shape of domain wall; the red arrows show the incident ports.

The schematic diagrams of wave propagation in the different phononic structures are demonstrated in Fig. 6 as a summary. The exchange of excitation ports (*e*.*g*., diagrams (1,1) and (2,1)), or the positions of topological and ordinary structures(*e*.*g*., diagrams (1,1) and (1,3)), can cause the reversion of the forward and backward transport of the elastic wave, which can be used to control the phonon transport along a specific path in phononic metamaterials. This selected phonon transport could be used to represent information, like the bits in conventional computing, providing a new way to design phononic logic gates.

In conclusion, by appropriately engineering of a pentamode structure, we have successfully constructed phononic structures in silicon with protected propagation of elastic waves which are immune to backscattering. The designed structures are characterized by pseudospin-dependent edge phonon transport. This approach yields a phononic metamaterial exhibiting helical edge states. It provides a specific lattice network topology where the phonon transport directions can be dynamically selected and controlled by changing configurations of structures. We want to emphasize that the one layer of patterning of our designed structures, the scalability of our results, and the mature fabrication processes available for silicon based systems render this an important possibility to fabricate tailored structures in different frequency regimes in particular higher frequencies reaching the GHz regime. Another key point of our work is the structure-dependent transport of elastic waves with topological protection. This propagation behaviour provides a further insight into the propagation possibilities. Besides the 2D pentamode structure investigated in this paper, it is expected other 2D or 3D novel structures will also exhibit similar topological features, which are waiting to be explored. Phononic helical edge states provide an intriguing way to fulfill the need of nonreciprocal elastic wave devices, open the door for nonlinear effects and active phononic metamaterials, and benefit the exploration of new forms of topological orders.

### Data availability statement

The datasets generated and analysed during the current study are available from the corresponding author on reasonable request.

## Electronic supplementary material

Supplementary information
