# Supplementary material for: Topological guiding of elastic waves in phononic metamaterials based on 2D pentamode structures
Source: Sci Rep. 2017 Dec 22;7:18043. doi: 10.1038/s41598-017-18394-8 (PMC5741736; doi:10.1038/s41598-017-18394-8)
Supplement: Supplementary file 1 — Supplementary information [file 41598_2017_18394_MOESM1_ESM.pdf]

Supplementary

Topological guiding of elastic waves in phononic metamaterials based on 2D pentamode structures

Yuning Guo[1,*], Thomas Dekorsy[1,2], and Mike Hettich[1]

[1] Department of Physics, University of Konstanz, 78457 Konstanz, Germany
[2] Institute of Technical Physics, German Aerospace Center, Pfaffenwaldring 38-40, 70568 Stuttgart, Germany
[*]corresponding. yuning.guo@uni-konstanz.de

1. Pentamode material

To show the "flow" ability of pentamode materials intuitively, the transmission of a pentamode structure in the fluid is demonstrated at a low megahertz frequency.

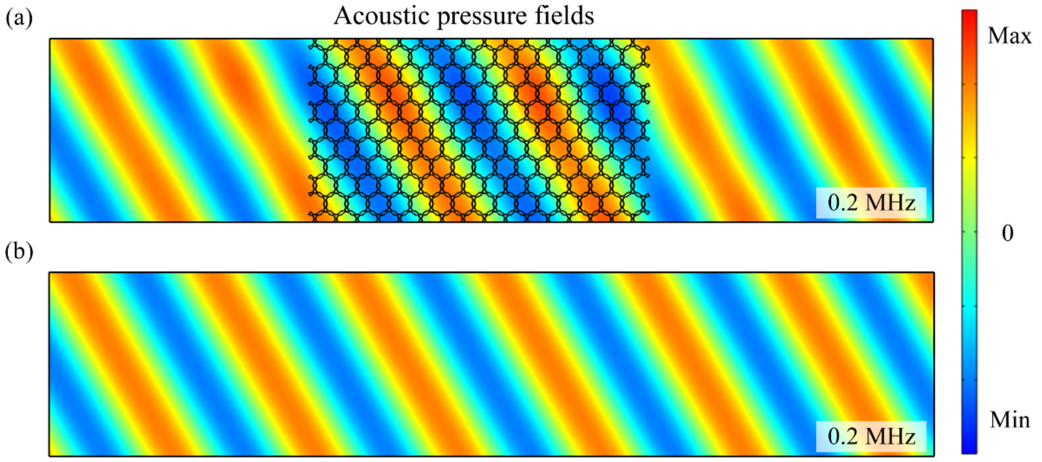

Figure S1. The acoustic pressure fields at 0.2 MHz with (a) plane wave incident on the pentamode structure of Silicon in the water; (b) the same plane wave incident on the water.

Figure S1 shows the acoustic pressure fields at 0.2 MHz with plane wave incident on the pentamode structure of Silicon in the water and the same plane wave incident on the water. The pentamode structure is the arrays of the unit cell with m=1.0. The plane wave with an incident angle of 30 degrees is set as background pressure field and the four outer boundaries are set as plane wave radiation. The density and sound speed of water are 1000 kg/m^3 and 1400 m/s, respectively. We can clearly observe that the existence of pentamode structure has little influence on the acoustic pressure field, *i.e.*, the pentamode structure can acts as acoustic fluids at this frequency. By changing the material parameters and the pentamode structure, the phononic properties of the solid structure are similar to that of the background medium, which keeps the propagating wave with the same waveform outside the area of the pentamode structure without obvious reflection on the side of the incident.

2. Berry curvatures of phononic structure

As Kramers doublet is applied in fermionic time-reversal symmetry, the Kramers degeneracy theorem cannot be applied directly for photon or phonon since they are bosons with spin -1. However, the physical explanations of time-reversal symmetry in the bosonic system have been established from other views. Bands with different topologies in dispersion relations cannot be transited into each other without closing the bandgap. Topological phase transition can take place at the domain wall after closing a trivial bandgap, neutralizing the Chern numbers and reopening a nontrivial bandgap. The Berry connection is $\mathcal{A}(k) = \langle u(k)|i\nabla_k|u(k)\rangle$ and the Berry curvature is $\mathcal{F}(\mathrm{k}) = \nabla_k \times \mathcal{A}(k)$. The Berry connection measures the local change of the phase of wavefunctions at a momentum space. The Chern number is calculated by integrating the Berry curvature over the Brillouin zone or analysing the symmetry of the lowest band at high symmetry points, which is $C=\frac{1}{2\pi}\oiint \mathcal{F}(k)\cdot \mathrm{d}s$. The Chern number characterizes the quantized collective behavior of the wavefunctions of bands, which can be considered as the number of monopoles of Berry flux inside a closed surface. It is the topological invariant of a 2D dispersion band that characterizes the non-trivial feature, which represents the winding number of the phase along the Brillouin zone boundary. When the Chern numbers of targeted bands are not zero, a topological photonic or phononic system with gapless edge states analogous to topological insulator emerges. The number of gapless edge states generally equals to the difference of the topological invariants across the interface[1].

The $k \cdot p$ method, which has been applied in photonic topological insulators, has also been used to calculate the Chern number of a phononic graphene structure. Here, k is the wave vector and p is the quantum mechanical

pulse operator. A double Dirac cone can be created with two double-fold states (pseudospin-up and pseudospin-down) in a bosonic system with artificially designed structures, which is analogue to the time-reversal symmetry of electrons. That guarantees the appearance of the Kramers doublet at Γ point of the C_6 symmetry structure[2,3].

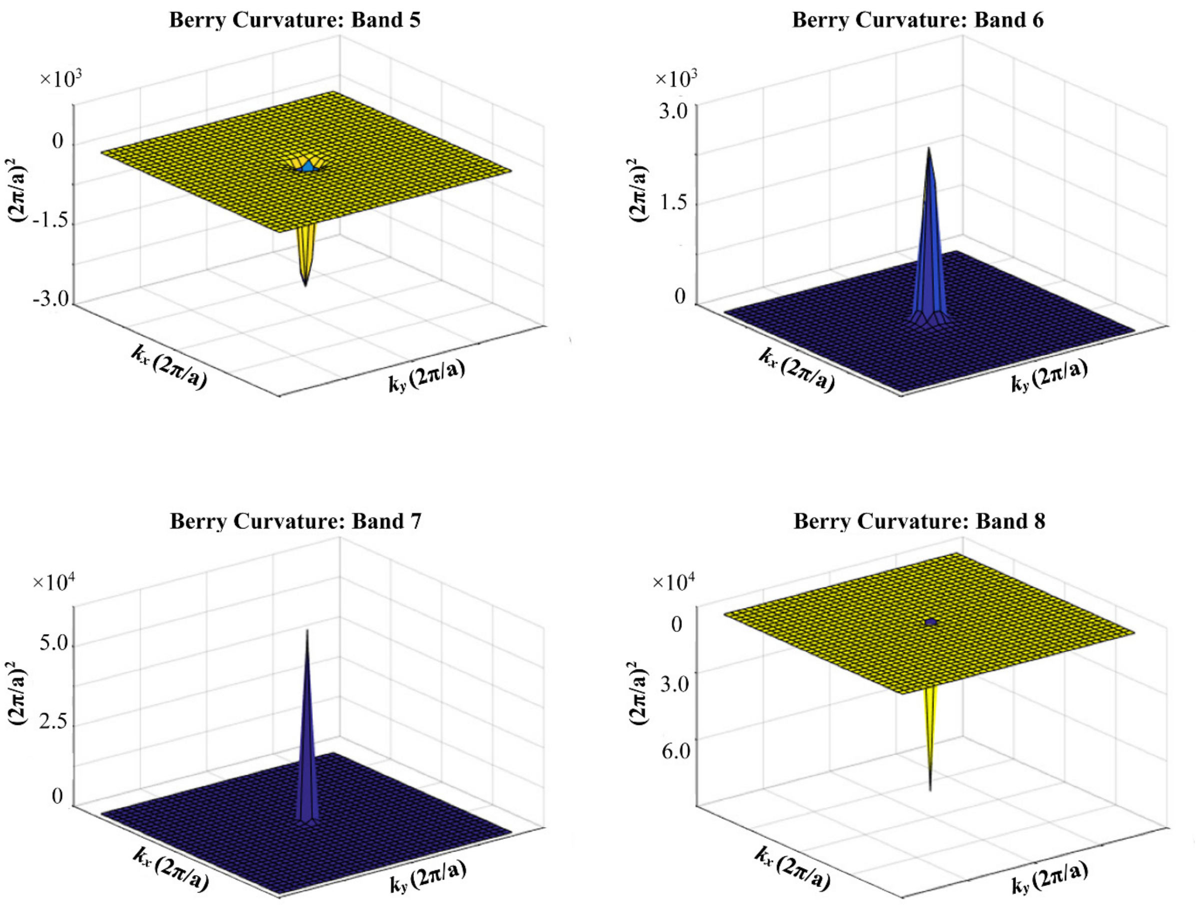

Figure S2. Berry curvature for the four bands (bands 5-8) above and below the nontrivial bandgap in the topological structure.

Each band can be characterized by a topological invariant. The Chern numbers can be obtained by integrating the Berry Curvature over the first Brillouin zone or analyzing the symmetry of the lowest band at high symmetry points[4]. A 9-point stencil in each dimension is used to calculate the Berry curvature. By using *Comsol linked with Matlab*, the Berry curvature of the phononic bands above and below the nontrivial bandgap in the reciprocal space are calculated as Figure S2 shows. The degenerate bands, *i.e.*, the band (5,6) and the band (7,8), have opposite signs of the Berry curvature, which is in accordance with the characteristics of the quantum spin Hall effect. When the integration of the Berry curvature near a peak or dip is performed, the Berry phase is equal to π or –π and the Chern number is $C_s=\pm1$. The topological transition appears with a band inversion between the bands (5,6) and (7,8) near the Γ point. The topological property of this phononic metamaterial is characterized by a non-zero Chern number of bulk bands, which implies that the edge states are preserved under continuous deformation.

According to the bulk-boundary correspondence principle, the number of edge states at a specific interface is decided by the change in the sum of the Chern numbers of the bulk bands at lower frequencies[5,6]. Since each Dirac point carries the Berry phase π, for one Dirac cone, the exchange of the Berry curvature between the two bands is 2π, which results in a chiral edge state in the gap [7]. Thereby, the helical edge states forms from the double Dirac cones. The Berry phase is independent of the edge states, which proves that it is the topology of bulk band structure that determines the topological property of a structure. By calculating the Chern numbers, we know that the bandgaps exhibit different topologies in different structures. To break the time-reversal symmetry, not only a nontrivial bandgap is needed, but also the topological transition point should be reached.

3. Protected propagation with disorders

Figure S3 shows the protected propagation of the phononic metamaterials with two kinds of disorders with the same frequency of 0.35 MHz as Fig. 3 and Fig. 4 in the paper. The top panels are the schematics and the bottom panels are the corresponding displacement contours. As expected both simulation results show a robust propagation despite the introduced disorder.

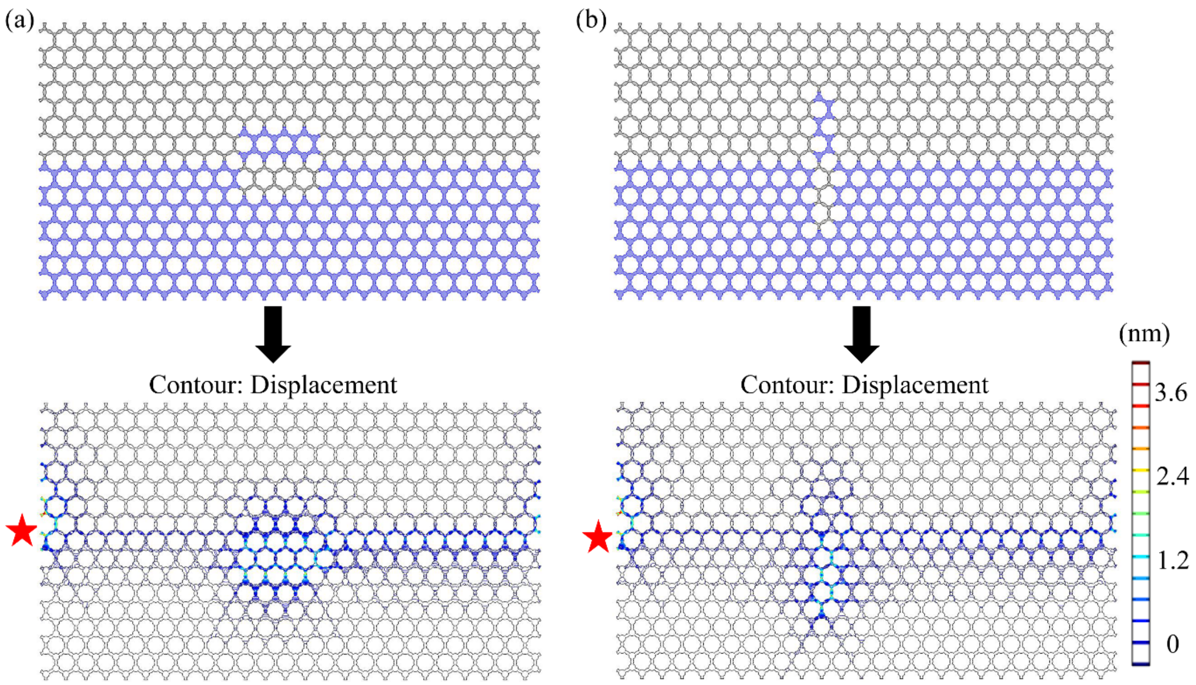

Figure S3. The protected propagation of the phononic metamaterials with two kinds of disorders with a frequency of 0.35 MHz. Top panels are the schematics and the bottom panels are the corresponding displacement contours.

References

1. Lu, L., Joannopoulos, J. D. & Soljacic, M. Topological photonics. *Nat. Photon.* **8**, 821-829 (2014).
2. He, C. *et al.* Acoustic topological insulator and robust one-way sound transport. *Nat. Phys.* **12**, 1124-1129 (2016).
3. Wu, L.-H. & Hu, X. Scheme for achieving a topological photonic crystal by using dielectric material. *Phys. Rev. Lett.* **114**, 223901 (2015).
4. Chen, W.-J. *et al.* Experimental realization of photonic topological insulator in a uniaxial metacrystal waveguide. *Nat. Comm.* **5** (2014).
5. Pasek, M. & Chong, Y. Network models of photonic Floquet topological insulators. *Phys. Rev. B* **89**, 075113 (2014).
6. Wang, Z., Chong, Y., Joannopoulos, J. D. & Soljacic, M. Observation of unidirectional backscattering-immune topological electromagnetic states. *Nature* **461**, 772-775 (2009).
7. Wang, P., Lu, L. & Bertoldi, K. Topological Phononic Crystals with One-Way Elastic Edge Waves. *Phys. Rev. Lett.* **115**, 104302 (2015).
